# Generation of Structural Components for Indoor Spaces from Point Clouds

**DOI:** 10.3390/s25103012

**Published:** 2025-05-10

**Authors:** Junhyuk Lee, Yutaka Ohtake, Takashi Nakano, Daisuke Sato

**Affiliations:** 1 School of Precision Engineering, The University of Tokyo, Tokyo 113-8654, Japan; lee@den.t.u-tokyo.ac.jp; 2 DataLabs, Inc., Tokyo 103-0024, Japan; takashi.nakano@datalabs.jp (T.N.); daisuke.sato@datalabs.jp (D.S.)

**Keywords:** 3D indoor modeling, 3D reconstruction, planar-based region growing, graph-cut, unsigned distance fields

## Abstract

Point clouds from laser scanners have been widely used in recent research on indoor modeling methods. Currently, particularly in data-driven modeling methods, data preprocessing for dividing structural components and nonstructural components is required before modeling. In this paper, we propose an indoor modeling method without the classification of structural and nonstructural components. A pre-mesh is generated for constructing the adjacency relations of point clouds, and plane components are extracted using planar-based region growing. Then, the distance fields of each plane are calculated, and voxel data referred to as a surface confidence map are obtained. Subsequently, the inside and outside of the indoor model are classified using a graph-cut algorithm. Finally, indoor models with watertight meshes are generated via dual contouring and mesh refinement. The experimental results showed that the point-to-mesh error ranged from approximately 2 mm to 50 mm depending on the dataset. Furthermore, completeness—measured as the proportion of original point-cloud data successfully reconstructed into the mesh—approached 1.0 for single-room datasets and reached around 0.95 for certain multiroom and synthetic datasets. These results demonstrate the effectiveness of the proposed method in automatically removing non-structural components and generating clean structural meshes.

## 1. Introduction

With recent advancements in laser scanners, increasingly accurate indoor scans have been reported. Consequently, the demand for the automatic reconstruction of indoor environments is increasing in emergency management [1,2], construction management [3], and physics simulations [4]. Various types of data, such as photos, depth images, and point clouds, are used as input data for indoor reconstruction. Point clouds are among the most commonly used types of data. However, three-dimensional (3D) indoor modeling using point clouds has challenges such as noise and occlusion owing to the presence of furniture and humans. Accordingly, 3D indoor modeling has been investigated via various approaches [5].

Addressing these problems is a fundamental issue for structural component generation. Ali et al. [6] introduced a reconstruction method based on a model-driven approach. After horizontally slicing the point clouds, the seed points located inside the model were extracted. The rectangular model was expanded from seed points to point clouds. The remaining parts were then reconstructed to create the entire model. However, this method cannot be used in multilevel indoor environments. Lim et al. [7] proposed a method that included piecewise plane extraction and energy minimization using a graph-cut algorithm. This method can be applied to multilevel buildings and multiroom environments. However, it requires trajectory data, including scan location information and preprocessing, to extract structural components.

The aforementioned issues are inevitably encountered when scanning actual spaces. Therefore, a global solution is required to address these complex issues.

In this paper, we propose an automatic 3D indoor structural generation method from point-cloud data, obviating the need for trajectory data and extracting structural point-cloud data. Our method only uses point clouds to generate a watertight indoor model. This approach was inspired by the watertight mesh-generation method proposed by Hornung et al. [8], which is used for mesh generation with non-uniform point-cloud data, such as point clouds with large void spaces. They utilized the unsigned distance function and the graph-cut algorithm. In our study, a pre-mesh is first generated from the entire point cloud to assign adjacency relationships to the data and compute the normal vectors of each vertex. Subsequently, plane meshes are extracted by the region-growing method. Next, pseudo-signed distance functions are generated using surface confidence estimation and a max-flow min-cut algorithm. Finally, a 3D indoor model is generated using a conventional surface reconstruction method and mesh flattening.

The proposed method is applicable to multilayer structures because it does not require decomposition or reconstruction. It is also a conventional approach that uses primitive extraction and graph cutting. Moreover, it is unnecessary to extract structural components because nonstructural components are automatically removed by inside/outside classification. In this study, we investigated a combination of voxel- and graph-based approaches. Representing the input data with voxel data as the distance field value in the intermediate process allows us to cope with noise. Subgraph structures based on voxel and graph-cut algorithms can remove indoor objects from interior spaces and fill in unscanned areas. Our method only requires point-cloud coordinate data, and there is no need to measure other data, such as the trajectory and color of the point clouds.

This remainder of this paper is organized as follows. Section 2 discusses relevant prior studies. In Section 3, we describe the proposed method. The experimental results and evaluations are presented in Section 4. Finally, Section 5 summarizes the conclusions drawn from this study and future research directions.

## 2. Related Work

Because point clouds obtained by a laser scanner inevitably encounter issues such as noise, scan errors, and occlusion due to the presence of furniture and humans, many methods have been investigated ways to address these problems [5,9]. Because indoor spaces are mostly composed of planes, such as walls, floors, and ceilings, one of the most representative methods for modeling indoor spaces is to extract and utilize these planes. Various methods for extracting planes, such as random sample consensus (RANSAC)-based or region-growing algorithms, have been reported.

This section describes previous work related to the proposed method. First, we present a review of the existing research related to mesh generation, followed by a description of the graph-cut algorithm and voxel-based approach in relation to the proposed method.

### 2.1. Surface Mesh Generation from Point Clouds

A fundamental method for converting point-cloud data into a mesh is the ball-pivoting algorithm [10]. This algorithm has the advantages of smooth surface generation, robustness to noise, and an effective representation of irregular structures. However, it is computationally complex and potentially degrades the mesh quality depending on the size of the sphere used. Another mesh-generation method is Poisson surface reconstruction [11]. This method generates smooth surfaces, even with noisy data, and achieves effective results with minimal parameter tuning. However, this method requires well-aligned, high-density point-cloud data and normal vectors for each point. These prerequisites highlight its limitations, as the accuracy and effectiveness depend heavily on the quality and arrangement of the input data. In this study, we generated so-called pre-mesh data, which were defined to establish adjacency relationships among point clouds. These data are fundamentally similar to mesh data but are specifically designed to only calculate the adjacency relationships and normal vectors required for plane extraction. Consequently, the orientation of the triangular meshes was not organized. For the pre-mesh, we utilized a spherical covering method [12]. This method efficiently generates meshes from irregularly distributed point clouds. The core idea involves using a spherical covering by placing spheres around each point to define localized support regions. This enables the parameterization of point data exclusively within the interior of each sphere centered on the points. This study defines and utilizes the unorganized mesh obtained during the intermediate process as the pre-mesh.

### 2.2. Plane Extraction

The most common approach for indoor modeling is based on primitive plane extraction. RANSAC is one of the most popular and effective methods for plane extraction in indoor point clouds owing to its robustness toward outliers and ease of implementation [13,14,15]. Li [13] proposed a plane-extraction method that uses normal distribution transform cells. The incorporation of normal distribution transformation (NDT) cells significantly enhances the RANSAC algorithm for 3D point-cloud plane segmentation. This approach improves robustness and accuracy by reducing spurious planes and streamlining plane detection, thereby increasing the efficiency of large-scale indoor point clouds. However, the careful tuning of NDT-specific parameters is required for optimal performance. Yang introduced an improved method for plane extraction in 3D point clouds using normal estimation and an optimized RANSAC algorithm [14]. This approach offers enhanced robustness by effectively handling noise and occlusions, improving accuracy through normal estimation and angular clustering, and increasing the efficiency suitable for large-scale datasets. However, it also introduces increased computational complexity and requires careful parameter tuning. Another study [15] achieved high accuracy and efficiency by projecting normal vectors onto a Gaussian sphere and optimizing high-order energy. This also ensures robustness and reliable segmentation using low-cost sensors. However, the complexity and dependence of this method on careful parameter tuning can be challenging. Similarly, the plane-extraction method using the RANSAC algorithm involves complex multistage algorithms that are challenging to implement. They require the careful tuning of various parameters for optimal performance, which can be time-consuming. Despite optimization, these methods remain computationally intensive, particularly for large-scale or detailed point-cloud data. There are also methods that utilize robust statistics [16]. This method effectively eliminates outliers, ensuring high robustness to noise and greater accuracy in complex environments. It reduces reliance on user-defined thresholds through automated parameter tuning and improves computational efficiency by eliminating iterative random sampling. However, the split-and-merge strategy increases computational costs, particularly in high-density data, and may struggle in cluttered indoor environments with intersecting planes, requiring additional refinement. While offering strong noise resistance, automation, and efficiency, its computational overhead and limitations in complex spaces suggest areas for further improvement. Another approach for plane extraction is the region-growing method. Region growing is a segmentation method used for data processing. It starts with a seed point and iteratively groups neighboring points that meet specific criteria, such as proximity and normal vector similarity, to form a region or plane. This method is effective in maintaining the continuity of surfaces and is intuitive to implement. If the adjacency relationship between the data can be established, region growing offers a simpler alternative for plane segmentation compared to RANSAC. Therefore, in the proposed method, we generate a pre-mesh to establish adjacency relationships within the data and calculate normal vectors that serve as search criteria. The pre-mesh, created temporarily for region growing, does not require the alignment of triangular mesh directions and can be a nonmanifold mesh.

### 2.3. Graph-Cut-Based Algorithm

Graph-cut algorithms based on planes extracted from point clouds have been widely used in indoor surface reconstruction [7,17,18,19]. Lim et al. [7] proposed an automatic indoor reconstruction system using piecewise plane extraction and energy minimization. This method is applicable to multistory buildings and indoor environments that are not included in Manhattan-world assumptions, such as sloped ceilings. However, it is necessary to record the scanning location based on trajectory data. Extracting object point clouds, such as furniture and humans, is also inconvenient. Some methods use graph cuts for horizontally sliced data [17,18]. These methods incorporate a regularization term into the energy function and generate a mesh with smooth surfaces. These approaches not only enhance the precision of wall segment extraction but also ensure the continuity and smoothness of the reconstructed surfaces, making them highly effective for detailed indoor surface reconstruction. Another approach focuses on reconstructing curved walls by extracting straight lines and curves from a sliced point cloud. Although this method omits certain corridor sections, it effectively creates the geometry of curved walls [19]. Many studies on indoor reconstruction using graph-cut techniques employ either horizontally sliced data or directly extract planes from 3D data. In the first case, applying this system to areas that do not conform to the Manhattan assumption, such as slanted walls, is challenging. In the second scenario, there is an additional process for separating indoor objects from structural elements in the input data. In this study, we use the voxel-based subgraph and graph-cut algorithm to generate a watertight mesh from the input point clouds while removing nonstructural elements without any additional preprocessing steps. Hübner et al. [20] proposed a geometrically complete indoor model generation method that represents a voxel. In this method, ceiling data are extracted from voxel data, and a model is generated based on these data. However, it cannot be applied to spaces beyond the Manhattan assumption and is unsuitable for complex indoor environments.

Ali et al. [6] used a model-driven approach for 3D indoor reconstruction. This method uses 2D sliced point-cloud data to extract seed points included in the model. The rectangular model is then expanded until it reaches the point clouds. Thereafter, the nonvertical parts of the rectangular model are adjusted, and a 3D indoor model is generated. However, this system is not suitable for multistory indoor models because of the difficulty in implementing sloped areas such as stairs. Therefore, it is applicable only to single-story models. Nonuniform point clouds are also unsuitable because they result in inappropriate 2D point-cloud slices.

## 3. Methods

**Figure 1:**
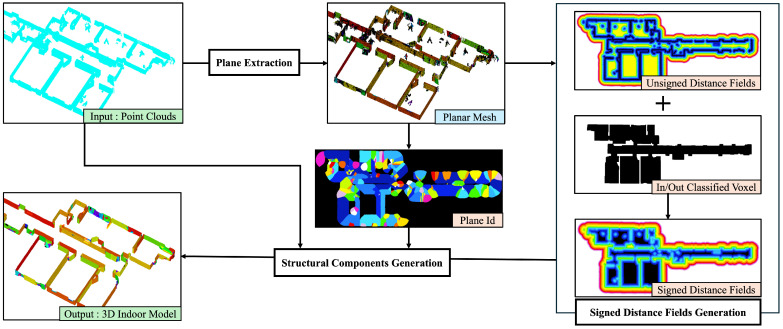
Flow of the proposed method.

In 3D indoor modeling, the extraction of nonstructural components is challenging. In recent research, data preprocessing is required prior to indoor model generation. This study realized inside/outside determination using the unsigned distance fields of structural components. No additional preprocessing is required for modeling the structural components. This section details the method for generating an indoor structural mesh, as shown in Figure 1. First, planar meshes are extracted based on a pre-mesh. Next, the signed distance fields of the plane meshes are calculated using the unsigned distance fields and the in/out classification. Finally, structural components are generated via dual contouring and mesh flattening.

### 3.1. Plane Extraction

**Figure 2:**
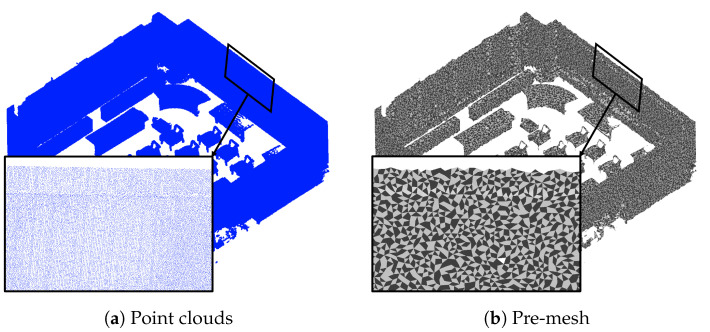
Pre-mesh generation.

In 3D indoor scenes, most of the structural components are planar. Therefore, the proposed method uses planar-based region growing for structural component extraction. However, extracting planes from noisy point clouds is complex. Therefore, we generate a pre-mesh to construct the adjacency relationship between each vertex and calculate the surface normal vectors. It is acceptable for the mesh to be unoriented, as shown in Figure 2, because the normal vector calculation does not align with the mesh. The spherical covering method [12] was used for pre-mesh generation. This is an effective method of generating meshes from noisy data.

We used planar-based region growing to extract the structural components. Using pre-mesh generation, we can calculate the vertex normal vector and obtain the adjacency relationship among the vertices. Plane extraction from the pre-mesh of the indoor space uses region growing with vertex normal vectors as extended criteria. Since the pre-mesh is not a clean mesh, applying region growing with a strict condition that requires the dot product of adjacent vertex normals to be exactly 1 makes planar segmentation difficult. Therefore, vertex normals with an angular difference of less than approximately 4 to 5 degrees were considered to be parallel through experiments. For this reason, the threshold was set to 0.997.

Because the pre-mesh exhibited rough surfaces, the vertex normal vectors calculated from the normal vectors of the triangular mesh did not align. This led to overclustering on a single plane, even when they belonged to the same surface. To address this issue, prior to region growing, vertex normals can be effectively smoothed to facilitate plane segmentation using edge-preserving normal smoothing.

(1) $$\begin{array}{cc} \; & n\left(P\right)\leftarrow \frac{\sum w_{i}n\left(Q_{i}\right)}{∥\sum w_{i}n\left(Q_{i}\right)∥}\\ \; & w_{i}=\mathrm{sign}\left(n\left(P\right)\cdot n\left(Q_{i}\right)\right)\cdot e^{-\frac{\rho_{i}^{2}}{c}}\\ \; & \rho_{i}=\frac{\arccos(|n\left(P\right)\cdot n\left(Q_{i}\right)|)}{∥PQ_{i}∥} \end{array}$$

**Figure 3:**
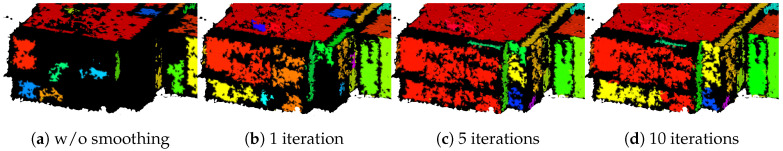
Experimental result for the determination of smoothing iterations.

As shown in Equation (1), the vertex normal vector n(P) is smoothed by the weighted sum of the normal at the vertex $Q_{i}$ adjacent to vertex *P*. The weight of each normal is calculated using the dot product $n\left(P\right)\cdot n\left(Q_{i}\right)$ of the angle and distance between vertices *P* and $Q_{i}$, respectively. This process was conducted iteratively on all vertices. To determine the appropriate number of iterations, normal smoothing was applied to an indoor model with varying iteration counts, and the resulting pre-mesh plane-extraction outputs were compared through labeling. Figure 3 shows the plane-extraction results corresponding to different numbers of normal smoothing iterations. Without applying normal smoothing, it was observed that the ceiling and the left wall regions are segmented into multiple labels. When the number of iterations was increased to 5, the occurrence of multiple labels on a single planar surface was reduced. However, further increasing the number of iterations led to the over-segmentation of planar regions, as shown in the left wall of Figure 3.

### 3.2. Signed Distance Field Generation

This section provides a detailed explanation of the process for generating a signed distance field (SDF). This process automatically removes unstructured parts within the interior, fills voids in structural parts that are not measured by LiDAR, and generates the SDF required for creating a watertight mesh.

**Figure 4:**
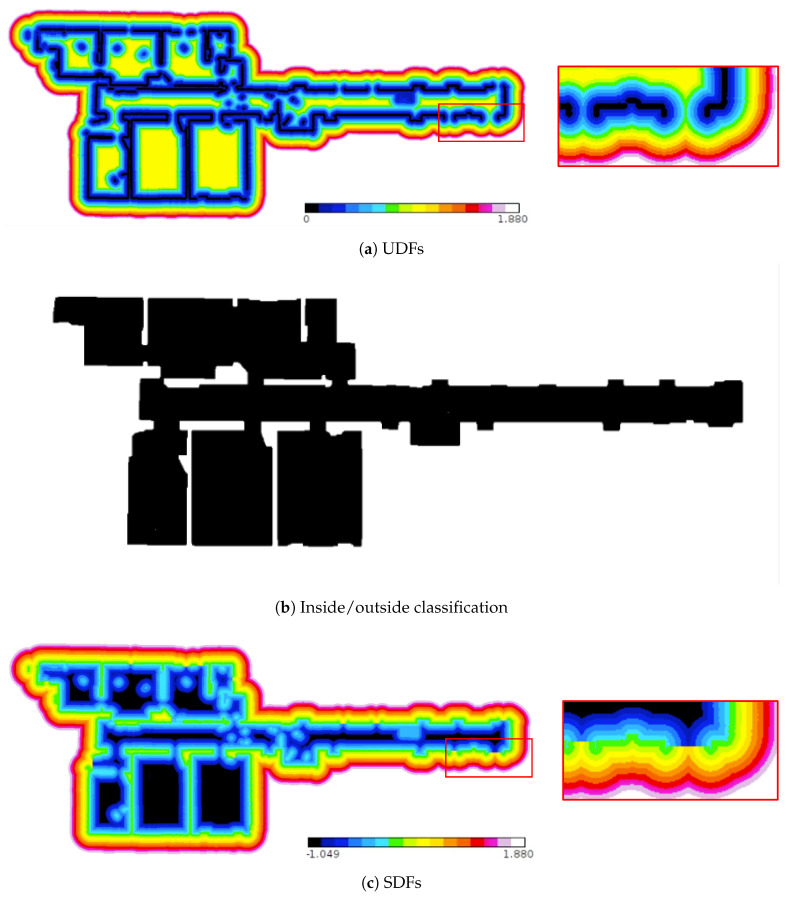
SDF generation: The SDF is generated as the product of the values stored in each grid of the UDF and inside/outside classification. In the red rectangle on the right-hand side of (**a**), there are two non-zero values of the distance function in the area that appears as a boundary between the inside and outside of the indoor model. However, in (**c**), compared to (**a**), the values of distance function near the same area at (**a**) are discontinuous but changed to zero.

The SDF is generated as follows:

**Step** **1:** The unsigned distance field (UDF) of the entire model is calculated (see Section 3.2.1 and Figure 4a)
  The UDFs of the plane meshes are then calculated. Subsequently, all UDFs are compared, and the minimum value is assigned as the distance value to generate the UDF for the entire model.
**Step** **2:** The inside and outside of the structural model are classified (see Section 3.2.2 and Figure 4b)
  Using the UDF of the entire model, a surface confidence map (voxel data) labeled with $V_{\mathrm{in}}$, $V_{\mathrm{out}}$, and $V_{\mathrm{crust}}$ is generated. A subgraph is then constructed based on $V_{\mathrm{crust}}$, and data distinguishing the interior and exterior regions of the indoor model are obtained by applying a graph-cut algorithm.
**Step** **3:** The SDF is generated (see Section 3.2.2 and Figure 4c)
  The SDF based on the surfaces of the indoor structures is generated using the UDF of the entire model obtained in Step1 and classification data derived in Step2.

#### 3.2.1. UDF Generation for the Entire Model

We first calculate the UDFs of each plane mesh before generating the UDF of the 3D indoor model. This approach was adopted to integrate the plane IDs and their corresponding normal vectors derived from plane extraction into the watertight mesh and mesh-flattening processes described in Section 3.3. The UDF of the indoor model is presented in Figure 4a. To reduce the computational load, we set a maximum value, calculated up to this value, and assigned a maximum value to the grids exceeding it. By selecting the minimum value of the UDFs at the overlapping voxels, individual UDFs can be effectively merged to generate a unified UDF that represents the indoor spatial model.

#### 3.2.2. Inside/Outside Classification

**Figure 5:**
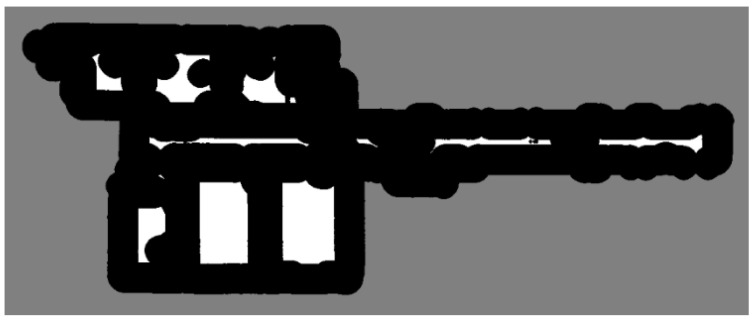
Surface confidence estimation: black is $V_{\mathrm{in}}$, gray is $V_{\mathrm{crust}}$, and white is $V_{\mathrm{out}}$. This figure shows a slice of voxel data created by TUB1.

After generating the UDF for the 3D indoor model, a surface confidence map was estimated based on the UDF value. Essentially, a surface exists when the distance is zero. However, the occlusion problem of the laser scanner results in an unscannable area, and its distance is nonzero. Therefore, we estimated the possibility of an existing surface. The voxels with a lower UDF value than the set maximum value were labeled as $V_{\mathrm{crust}}$. As a result, we created a voxel divided into three regions: inside the indoor model $V_{\mathrm{in}}$, outside the indoor model $V_{\mathrm{out}}$, and a high possibility region of a surface $V_{\mathrm{crust}}$. In Figure 5, the three regions are depicted using three different colors.

After plane extraction, nonstructural components with flat surfaces were labeled, and $V_{\mathrm{crust}}$ contained both the structural and nonstructural components. The max-flow min-cut algorithm was used to classify the inside and outside of 3D indoor model and remove nonstructural components. The max-flow min-cut algorithm is a graph-cut algorithm that divides a graph into two sets by splitting the edges into the smallest sum of weighted edges. This algorithm was implemented by minimizing the energy function described in Equation (2). Graph *G* is based on the distance function of the 3D indoor model. *G* has a six-neighborhood structure of the grid structure in $V_{\mathrm{crust}}$. All nodes included in the outside grids $V_{\mathrm{out}}$ are connected to the sink node, and all nodes included in the inside grids $V_{\mathrm{in}}$ are connected to the source node. Thus, the graph of the nonstructural components in the indoor model is only connected to the source node. As a result, the nonstructural components were automatically removed after graph cutting. We obtained boundary *B* between the inside and outside grids by splitting the edges, *e*, because they are set by the connection of two adjacent grids.

(2) $$E\left(B\right)=\sum_{v_{i},v_{j}\in B}w\left(v_{i},v_{j}\right)$$

In this study, we set the weight function based on the distance function value described in Equation (3). Essentially, a surface exists when the distance is zero. By cutting the edges that minimize the value of this function, we can find two grids where the surface exists. The boundary between these two grids becomes the criterion for classifying the interior and exterior of a 3D indoor model. In previous studies [21], they set s=4 and $a=1.0\times 10^{-5}$. After experiments with various formulations and parameter settings, we adopted the same equation and parameter with previous studies. This will be discussed in detail in Section 4.3.

(3) $$w\left(v_{i},v_{j}\right)={\left(\frac{\phi \left(v_{i}\right)+\phi \left(v_{j}\right)}{2}\right)}^{s}+a$$

**Figure 6:**
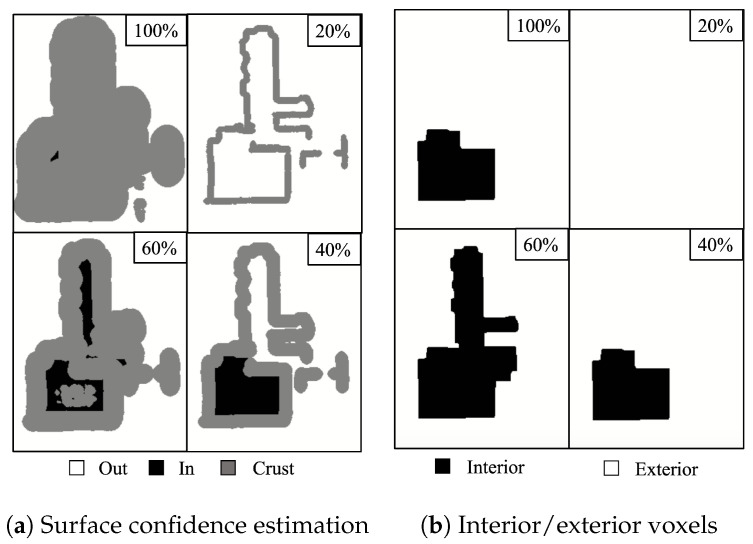
Results of inside and outside classification using surface confidence estimation.

Depending on the set maximum distance value, $V_{\mathrm{crust}}$ may not play a role in dividing the inside and outside grids, resulting in the regions not being divided into three, as shown in Figure 6. Therefore, we first set the maximum distance to be as large as possible. We then decided to sequentially decrease the maximum value to estimate surface confidence. Subsequently, we selected the value that divided the largest number of inside grids when the max-flow min-cut algorithm was applied. Because the distance between the ceiling and the floor in single-level indoor environment usually does not exceed 3 m, we initially set the maximum distance value to 1.8 m and considered filling the unscanned area. By assigning a value of −1 to the voxels labeled as “inside’’ and +1 to those labeled as “outside’’ from the classification data obtained in the preceding steps and multiplying these values by the UDF value of each voxel in the entire model, the resulting SDF delineates the boundaries of structural components while assigning negative values to all unstructured components.

### 3.3. Generation of Structural Components

**Figure 7:**
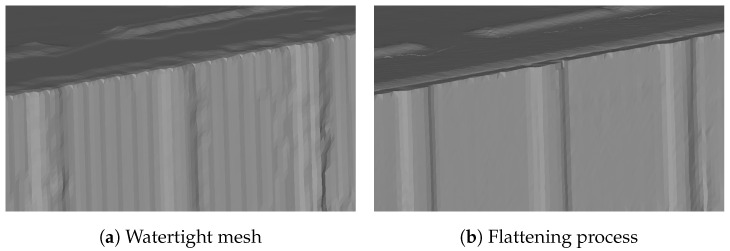
Flattening process.

This section describes two processes: watertight mesh generation and mesh flattening. A watertight mesh can be generated using dual contouring [22]. As explained in Section 3.2, the generated SDF assigns negative values to all unstructured components. Therefore, by extracting the iso-surface and constructing the mesh through this process, a mesh containing only the structural components can be obtained. However, as shown in the result on the left-hand side of Figure 7, there is an issue where flat surfaces are inaccurately represented with an uneven appearance. Therefore, a mesh-flattening process is necessary to produce a model with smooth and clean planar surfaces, as shown on the right-hand side of Figure 7.

**Figure 8:**
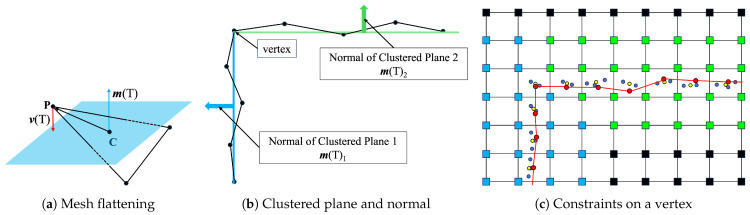
Mesh flattening based on plane extraction.

**Figure 9:**
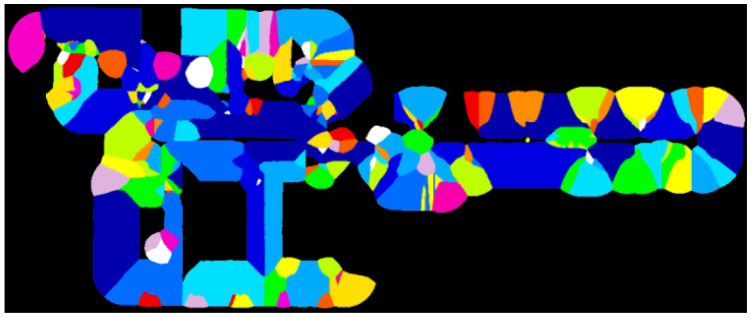
Plane ID: This is used to associate the watertight mesh and plane IDs, which are the calculated normal vectors from the plane-extraction process. The colors of the Plane ID are surface labels.

We performed the surface flattening of the rough mesh based on clustered planes, as illustrated in Figure 8. As shown in the center of Figure 8, the vertices of the watertight mesh are updated based on the labels obtained during the division of the pre-mesh and normal vectors of the segmented planes. These normal vectors from label are abtained from Plane ID (Figure 9). However, relying on the normal vectors of the segmented planes may result in a misalignment with the actual point clouds when iteratively updating the vertices. To address this issue, as shown on the right-hand side of Figure 8, because dual contouring generates vertices in the cell, the vertices of the watertight mesh generated through the graph cut are constrained to the average coordinates of the actual point clouds existing within the same cell. This constraint ensures that the flattened mesh aligns closely with the actual point clouds, thereby minimizing discrepancies. Examining Equation (4) and the left-hand side of Figure 8, there are three types of vertices or points: $P_{\mathrm{old}}$ is the vertices of the watertight mesh before updating, $P_{\mathrm{new}}$ is the vertex of the mesh updated using the point-cloud coordinates as constraints, and $P_{\mathrm{origin}}$ is the average point-cloud coordinate for each voxel. α is a user-specific parameter. In this study, we set this parameter to 0.1. By considering the area A(T) of the adjacent triangle mesh *T* with vertex P, the distance from P to the center C of the triangle, and the average normal vector m(T) of the segmented mesh, a flattened mesh can be generated by iteratively by updating all vertices. In addition, because the occlusion of point clouds does not generate a pre-mesh, there are no labels on the watertight mesh. This area was flattened using Laplacian smoothing.

(4) $$\begin{array}{c} \left\{\begin{array}{c} P_{\mathrm{new}}\leftarrow \alpha P_{\mathrm{origin}}+\left(1-\alpha \right)\left(P_{\mathrm{old}}+\frac{1}{A(T)}\sum A\left(T\right)v\left(T\right)\right)\\ v(T)=[(C-P)\cdot m(T)]m(T) \end{array}\right. \end{array}$$

## 4. Results

In this section, we evaluate the proposed method for use with two real-world datasets including two datasets collected by us, two datasets from the ISPRS benchmark datasets, and two synthesized datasets. We acquired data from a real-world indoor environment using Leica BLK360, labeled as A and B. These are single-room models. Additionally, we used two of the ISPRS benchmark datasets on indoor modeling [23]: TUB1 and TUB2. These are multiroom models, and TUB2 is a multifloor model. Two synthesized models from the UZH dataset [24], syn1 and syn2, were also used in the experiment. The previous four models were based on the Manhattan-world assumption, whereas these two models did not follow this assumption. The scanners used to obtain the dataset and information on the indoor environment are listed in Table 1. The first two models were single-room models evaluated by measuring the distance between the mesh created using the proposed method and the input data point cloud.

### 4.1. Evaluation

This section describes the evaluation metric used to assess the proposed method. The first evaluation metric is the mean distance error, which is defined as the average of the signed distances from the input point cloud to the output structural components. In this study, the colormap of the point-to-mesh signed distance was generated using CloudCompare [25]. The second evaluation metric is completeness, which quantifies how much of the original point cloud has been successfully reconstructed into the mesh. It is defined and computed as follows:

(5) $$\mathrm{Completeness}=\frac{\left|\{\;q_{\mathrm{pts}}|\exists \;p_{\mathrm{mesh}}:\;∥q_{\mathrm{pts}}-p_{\mathrm{mesh}}∥\leq \tau \}\right|}{N_{\mathrm{pts}}}$$

For each input point $q_{\mathrm{pts}}$, we check whether there exists at vertex $p_{\mathrm{mesh}}$ on the evaluated mesh surface that lies within a distance threshold τ. The points satisfying this condition are counted, and completeness is expressed as the ratio of this count to the total number of input points $N_{\mathrm{pts}}$.

### 4.2. Structural Component Generation Results

In this section, we describe the experimental parameters. To ensure consistency in the experiment, the parameters used for pre-mesh generation, plane segmentation using region growing, and mesh flattening were standardized across all models. However, the voxel pitch varies depending on the model (Table 2). In Models A and B, which are single-room models, the spatial structure is simpler than that of the other models. Therefore, as listed in Table 2, a larger voxel pitch was set to reduce the execution time. For other models that include multiple rooms, considering the distance between the walls of the rooms is essential. Therefore, the voxel pitch was set to a smaller size compared with Models A and B. When applying the proposed method to multiroom or synthesized models, reducing the voxel pitch allows for a finer representation of thin walls and narrow inter-floor gaps, thereby enhancing the overall geometric fidelity of the reconstructed mesh. Accordingly, in this experiment, the voxel pitch was set to the lowest value that could be processed within the available computational resources. Additionally, considering that the typical height from the floor to the ceiling in indoor environments ranges from approximately lower than 3.9 m [26], the max distance value was set to 1.8 m. This setting ensures the adequate coverage of unmeasured regions that may arise during LiDAR-based indoor scanning, such as occlusions near ceilings or under furniture.

**Figure 10:**
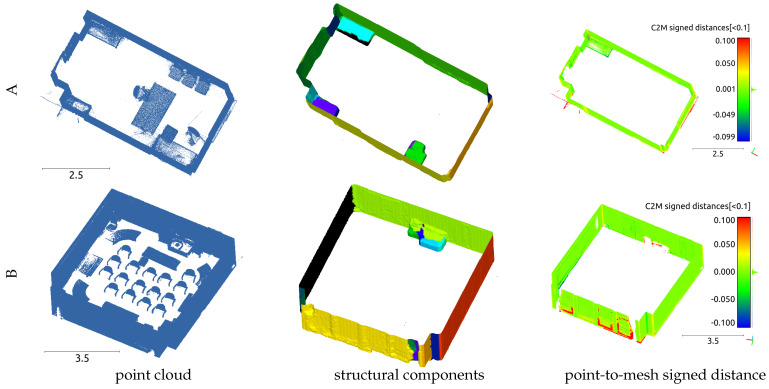
Single-room models: visualizations of point clouds, structural components, and evaluation result for (**A**,**B**).

First, we present the experimental results for the single-room models in Figure 10. Evidently, the objects present in the indoor space have been removed, leaving the structural components meshed. However, the three small bookshelves attached to the walls were not removed. The proposed method fundamentally extracts planes and calculates the UDF based on these planes. When the outside and inside of the model were distinguished using graph cuts, indoor objects were automatically assumed to be removed. Therefore, objects with planar structures attached to walls cannot be separated. The color map in Figure 10, which shows the distance between the mesh and the point cloud, indicates that the meshes were successfully generated for all structural components. In the color map of Model B, the red-colored areas correspond to curtains, which are curved surfaces. However, during plane segmentation, these areas are treated as planes because of the settings of the region-growing algorithm, which uses the dot product of the normals of the adjacent mesh vertices as a search condition. Consequently, these areas were smoothed during the mesh-flattening process, resulting in significant discrepancies from the actual data. The means and standard deviations of the distances between the datasets and output data are presented in Table 3. When comparing point-cloud and watertight mesh data, the mean distance errors were 7.6 and 5.6 mm, respectively. In contrast, when comparing the final data generated through flattening, the mean errors were reduced to 3.9 and 2.7 mm, respectively, indicating that mesh-flattening results in more precise data. This result is also reflected in the standard deviation values.

**Figure 11:**
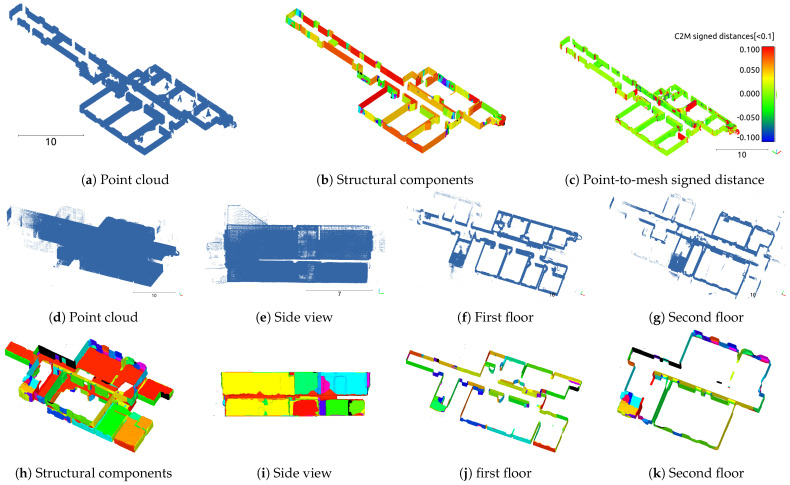
Multiroom models: visualizations of point clouds, structural components, and evaluation results for TUB1 and TUB2.

Next, we analyze the results obtained from the multiroom model. Considering the TUB1 results shown in Figure 11, a comparison between the point-cloud data obtained through LiDAR and the output mesh data generated by the proposed method revealed that some of the walls were not properly reconstructed. This tendency was more distinct in the results obtained from the TUB2 dataset. In this case, representing a multilevel indoor space obtained through actual measurements, the proposed method generated a mesh without a clear separation between the first and second floors. Consequently, as shown in Table 3, the TUB2 dataset exhibited a significant error of −76.1 mm when comparing the distance between the point cloud and watertight mesh. In contrast, the TUB1 dataset showed a distance error of −13.1 mm for the watertight mesh, which further decreased to −10.5 mm after applying mesh flattening, indicating a relatively small error, similarly to the single-room model.

**Figure 12:**
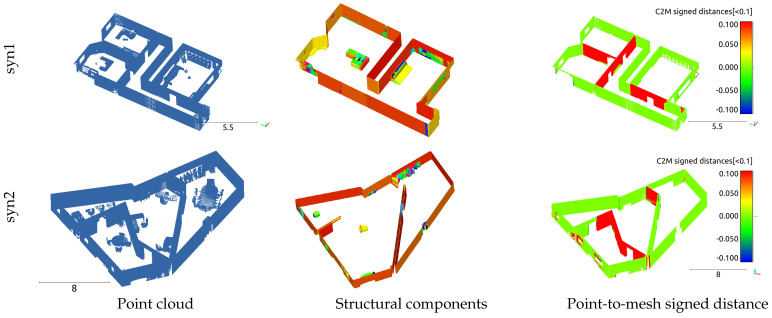
Synthesized models: visualizations of point clouds, structural components, and evaluation result for syn1 and syn2.

Finally, to assess the applicability of our method to datasets that do not adhere to the Manhattan-world assumption, the developed method was applied to two synthesized datasets. The corresponding results are shown in Figure 12. In this case, similarly to the above results, all nonstructural components, except for those fully attached to walls or floors, have evidently been removed, resulting in the generation of a mesh representing only the structural components. Additionally, the meshes of walls that were not aligned with the orthogonal coordinate system were also successfully generated. However, the inspection of the watertight mesh and distance error color map in Figure 12 reveals that, similarly to the multiroom model, certain wall surfaces were not fully generated. As a result, the error values for the watertight mesh were 61.4 and −36.2 mm, which were larger compared to other cases. However, the final generated data showed a reduction in the error values to 52.7 and −27.2 mm. Consequently, across all datasets, the mesh-flattening process effectively reduced the error in the final generated data. By applying the proposed method to reconstruct indoor spaces for single-room, multiroom, and non-Manhattan-world-assumption models, we successfully generated the meshes of structural components, while excluding nonstructural components, using only point-cloud data. For most reconstructed indoor spaces, such as Models A, B, and TUB1, the error in the generated meshes ranged from approximately 2 to 10 mm. However, in certain indoor spaces, such as TUB2, syn1, and syn2, some walls and floors were not properly formed.

**Figure 13:**
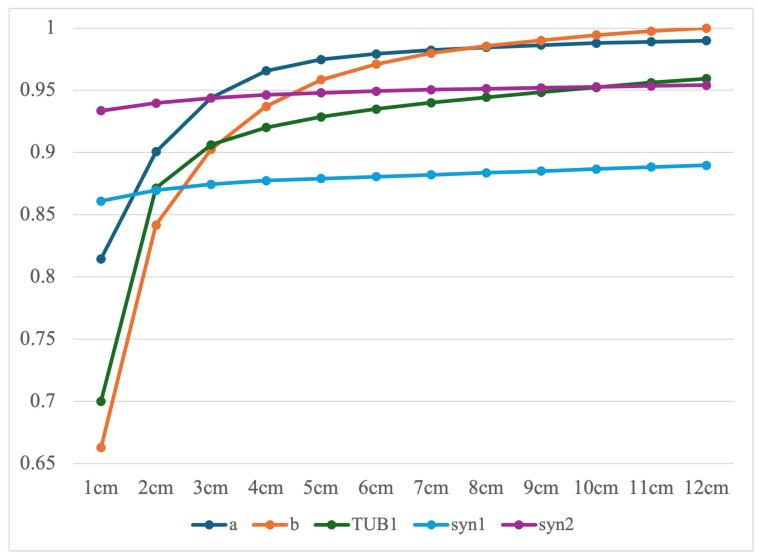
Results of completeness.

As the next evaluation, we investigate the completeness values for each dataset individually. Excluding TUB2, which did not produce structural components due to significant reconstruction errors, we computed the completeness metric for the remaining five datasets. The threshold τ, as defined in Section 4.1, was varied from 1 cm to 12 cm. The resulting completeness values were calculated for each dataset and are presented in Figure 13. For five datasets, the completeness values converge at a threshold τ of 5 cm, with values exceeding 0.85. In particular, the completeness for single-room datasets approaches 1.0, while that for multiroom datasets is close to 0.95. This indicates that the proposed method is highly effective for single-room environments and remains effective even in more complex multiroom environments.

### 4.3. Discussion

**Figure 14:**
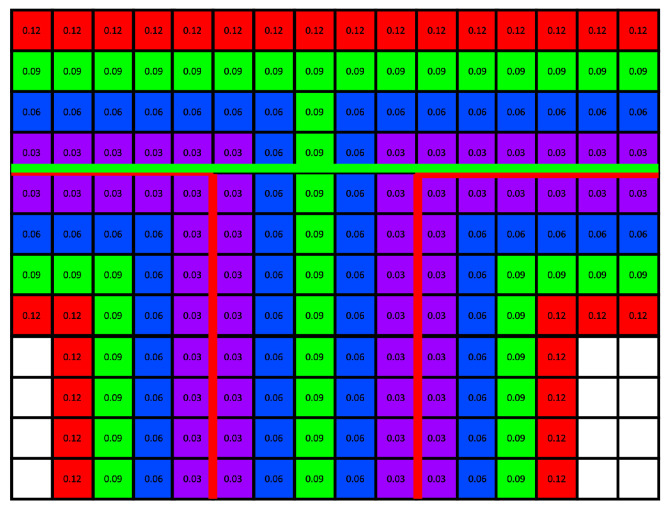
Example of the graph-cut problem.: A simplified model was created to provide a visual representation of the challenges encountered in the graph cut.

Figure 14 shows a cross-sectional portion of the UDF with a voxel size of 0.03, created to explain these issues in detail. The distance from the surface is indicated by the color gradient transitioning from purple to red as the distance increases. In Figure 14, the purple voxels represent those closest to the surface. Ideally, the graph cut should distinguish the inside and outside of the model based on the red line. However, the experimental results show that separation occurred along the green line, which likely explains why some walls and floors were not properly generated. This is attributable to the function used to calculate the edge weight in the graph, which may not accurately reflect the desired separation criteria. The sum of the edge costs using Equation (3) along the red and green lines was calculated using the UDF values shown in the figure. The sum of the edge costs along the red line was approximately $2.7\times 10^{-3}$, whereas the sum along the green line was $1.6\times 10^{-3}$. Because the green line is shorter, the subgraph constructed based on the voxels is cut along the green line, resulting in the observed segmentation.

**Figure 15:**
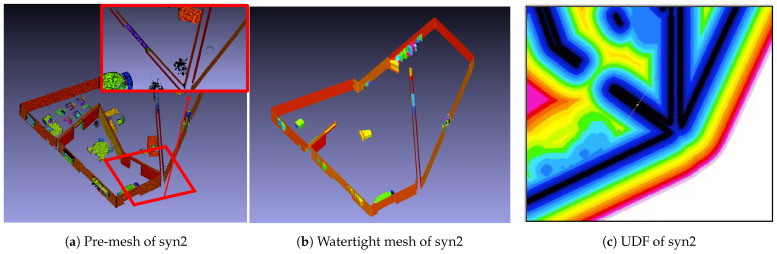
Graph-cut problem with experiments.

**Figure 16:**
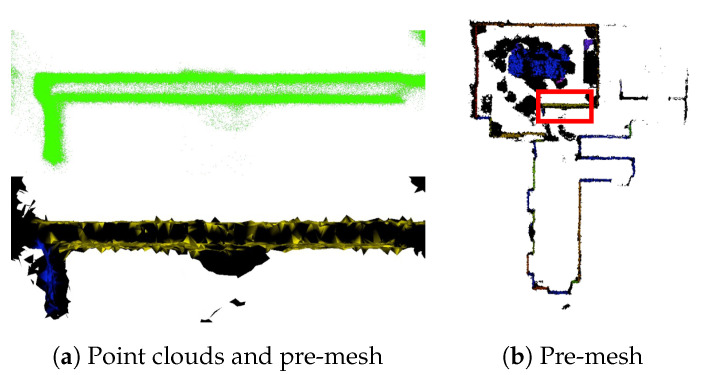
Pre-mesh and watertight mesh (the pre-mesh represents a top–down view of the room model with the meshes of the ceiling and the floor removed).

In the pre-mesh data shown in Figure 15, viewing the cross-section from above reveals the presence of two walls. When the proposed method was applied to generate the final mesh, the left wall was not reconstructed, and the right wall was successfully generated. Moreover, there are other limitations to this method. As shown in red box on the right-hand side of Figure 16, during the process of generating a pre-mesh, there is an issue where two planar meshes are represented as a single mesh. This refers to areas where walls are measured using LiDAR from both the room and hallway, resulting in the presence of two separate meshes. When multiple rooms exist within an indoor space, the recognition of multiple segmented spaces separated by walls as a single space is limited. It is predicted that this recognition limitation occurs when the walls are thin and a single mesh is generated, resulting in their disappearance during the watertight process. The graph-cut algorithm used to separate the interior and exterior of the model, as described in Section 3.2, is implemented by solving an energy minimization problem. When discretized, the energy function can be represented as shown in Equations (2) and (3). In these equations, the term ${\left(\frac{\phi \left(v_{i}\right)+\phi \left(v_{j}\right)}{2}\right)}^{s}$ in Equation (3) corresponds to the data term, which encourages alignment between the distance field and the input point cloud. The term denoted as *a* is the smoothness term, penalizing overly complex or irregular surfaces.Through experimentation, various functional forms were tested for the data term, in which the cost remains very low near zero-valued distance field regions and increases exponentially beyond a certain threshold. However, these alternatives showed negligible improvement over the formulation and parameter values used in prior work [8,21]. Consequently, we adopted the same function and parameters as those previously proposed.For this reason, as illustrated in Figure 14 and Figure 15, defining the data term using distance fields is shown to be insufficient—particularly when two or more large planar surfaces are located in very close proximity. Under such conditions, an ideal graph cut on the voxel-based graph becomes infeasible.

**Figure 17:**
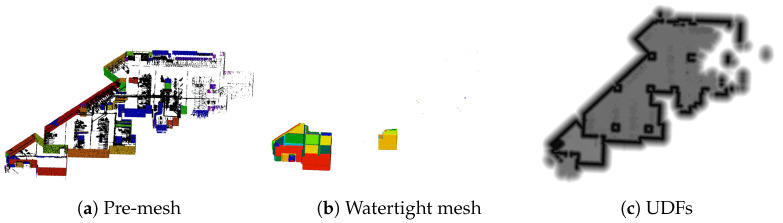
Pre-mesh, watertight mesh, and UDFs (the pre-mesh represents a top–down view without the ceiling and the floor).

Another restriction of the proposed method is shown in Figure 17. When generating a pre-mesh from point clouds, a mesh was generated regardless of the presence of labels. When generating a watertight mesh, only part of the model can be reproduced. This is illustrated in Figure 17. When performing the inside–outside determination to create a watertight mesh, if the holes in the original scanned point-cloud data are large, it becomes difficult to calculate the distance field up to the surrounding area of the surface. Consequently, these inside and outside classifications are not effective, and only a part of the model is displayed.

## 5. Conclusions

With the growing societal demand for indoor spatial data, corresponding research efforts have been actively pursued. This study aimed to generate structural components from LiDAR-derived point-cloud data by removing non-structural components, without the need for additional data separation processes. Our proposed method facilitated the generation of a pre-mesh using the sphere-covering method from the point clouds of an indoor space obtained by LiDAR. After undergoing edge-preserving normal smoothing, the generated mesh was then segmented into planes using region growing. Subsequently, by calculating the distance field, performing graph cuts, and using dual contouring, we generated structural components with watertight surfaces. After performing mesh flattening based on plane extraction, we obtained the indoor structural components. The experimental results demonstrated that this method generates structural components with watertight and flat surfaces, excluding nonstructural components such as furniture and people within indoor spaces.

## Figures and Tables

**Table 1:** Experimental datasets.

Datasets	Scanner	Rooms	Floors	Manhattan Assumption
A	BLK360	1	1	◯
B	BLK360	1	1	◯
TUB1	Viametris iMS3D	10	1	◯
TUB2	Zeb-Revo	14	2	◯
syn1	Synthesized	3	1	△
syn2	Synthesized	7	1	×

**Table 2:** Experimental parameters.

Datasets	Voxel Pitch (m)	Volume Size
A	0.05	178 × 119 × 101
B	0.05	211 × 212 × 108
TUB1	0.03	639 × 1532 × 216
TUB2	0.04	1147 × 660 × 310
syn1	0.03	380 × 601 × 216
syn2	0.03	862 × 852 × 249

**Table 3:** Results of the mean distance error.

	A		B		TUB1		TUB2		syn1		syn2	
	m	σ	m	σ	m	σ	m	σ	m	σ	m	σ
Watertight Mesh (mm)	7.6	34.7	5.6	41.6	−13.1	87.7	−76.1	545.1	61.4	197.4	−36.2	182.0
Structural Components (mm)	3.9	29.9	2.7	37.8	−10.5	84.7	0	0	52.7	195.6	−27.2	183.9

## Data Availability

The final results (3D models) obtained in this study are available upon request from the first author.

## Abbreviations

The following abbreviations are used in this manuscript:

UDF	Unsigned distance function;
SDF	Signed distance function.
